# Supplementary material for: Proteomic Analysis of Dupuytren’s Contracture-Derived Sweat Glands Revealed the Synthesis of Connective Tissue Growth Factor and Initiation of Epithelial-Mesenchymal Transition as Major Pathogenetic Events
Source: Int J Mol Sci. 2023 Jan 5;24(2):1081. doi: 10.3390/ijms24021081 (PMC9866571; doi:10.3390/ijms24021081)
Supplement: Supplementary file 1 [file ijms-24-01081-s001.zip › supplementary material.pptx]

## Slide 1
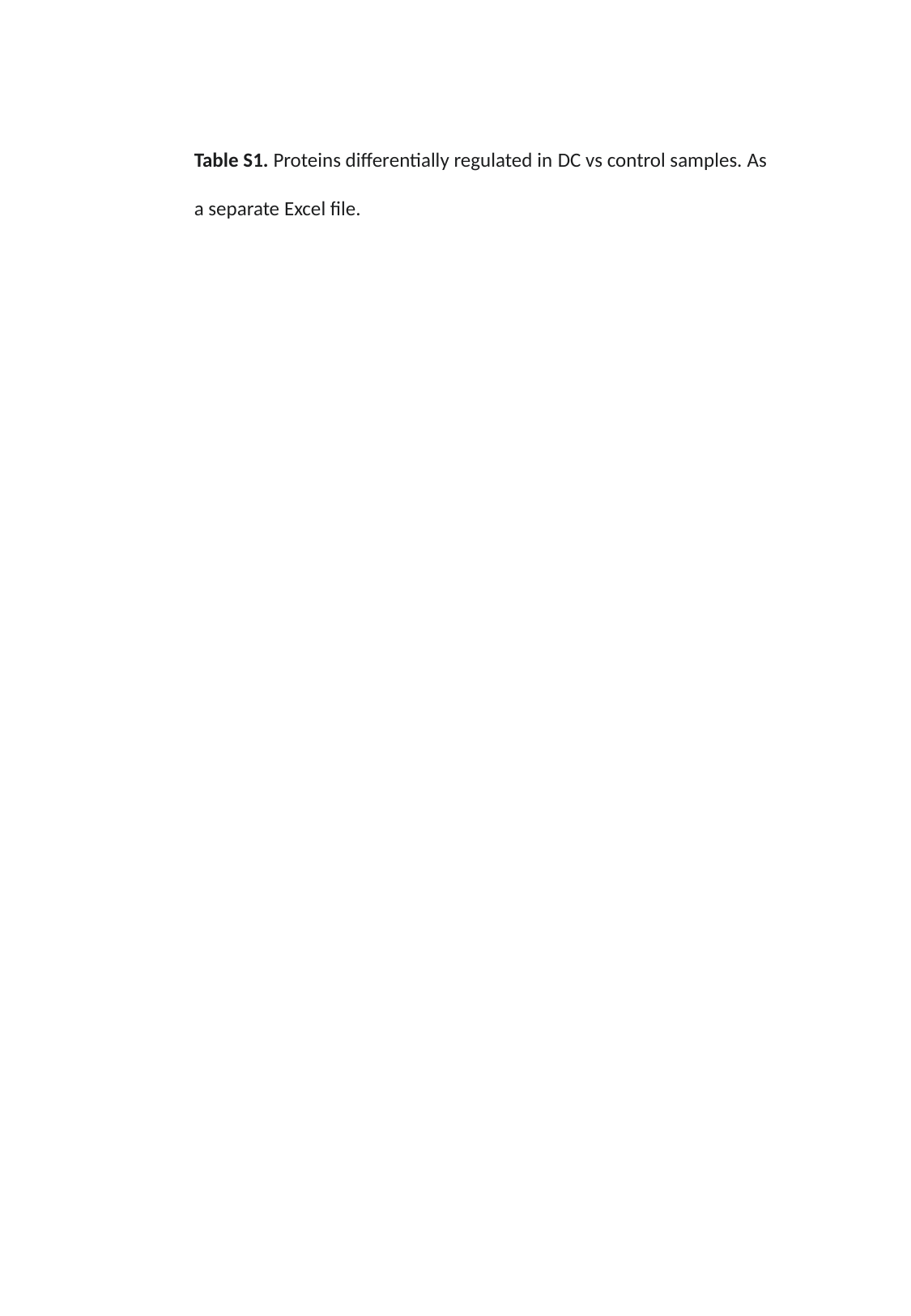

Table S1. Proteins differentially regulated in DC vs control samples. As a separate Excel file.

## Slide 2
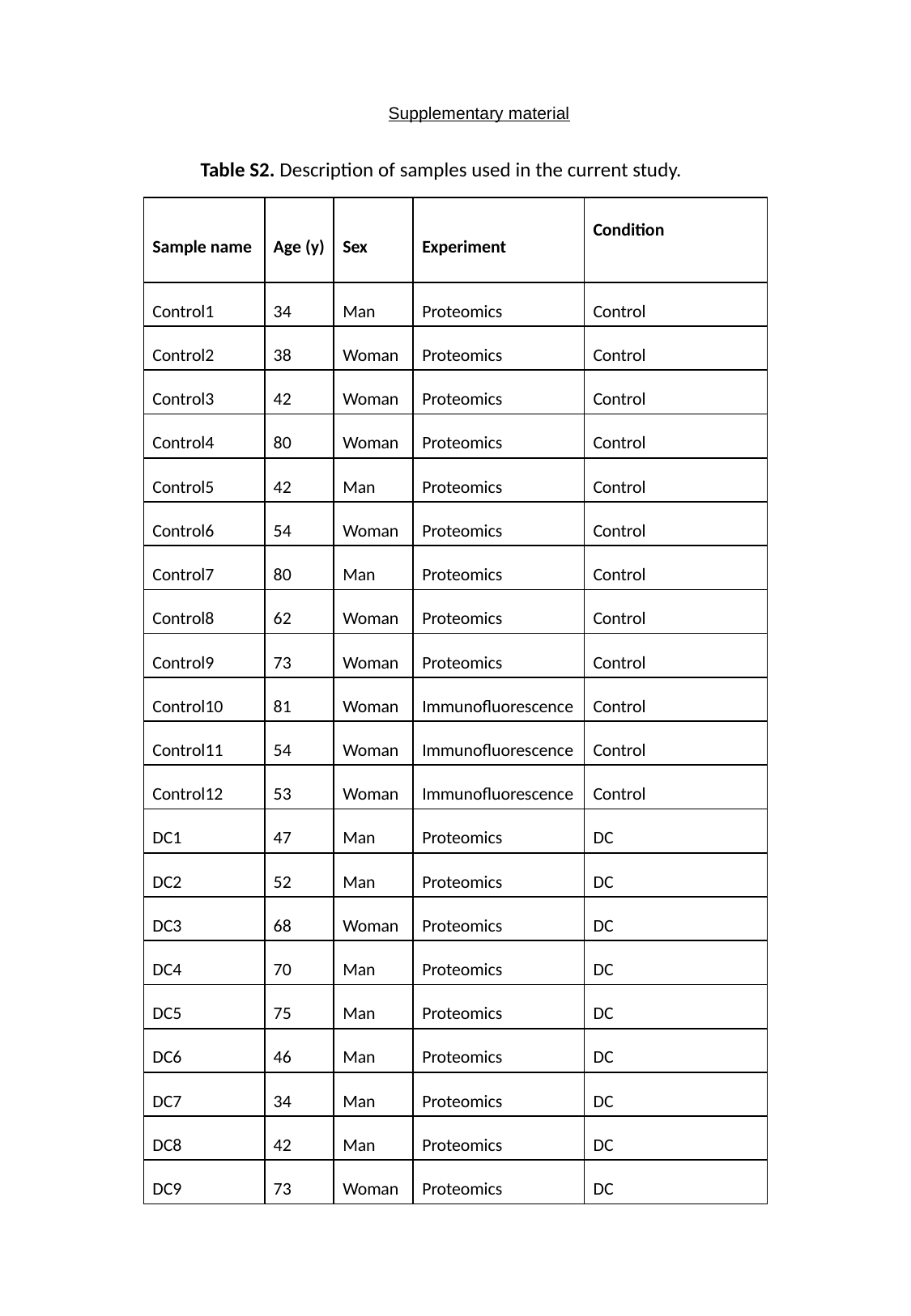

Supplementary material
Table S2. Description of samples used in the current study.
| Sample name | Age (y) | Sex | Experiment | Condition |
| --- | --- | --- | --- | --- |
| Control1 | 34 | Man | Proteomics | Control |
| Control2 | 38 | Woman | Proteomics | Control |
| Control3 | 42 | Woman | Proteomics | Control |
| Control4 | 80 | Woman | Proteomics | Control |
| Control5 | 42 | Man | Proteomics | Control |
| Control6 | 54 | Woman | Proteomics | Control |
| Control7 | 80 | Man | Proteomics | Control |
| Control8 | 62 | Woman | Proteomics | Control |
| Control9 | 73 | Woman | Proteomics | Control |
| Control10 | 81 | Woman | Immunofluorescence | Control |
| Control11 | 54 | Woman | Immunofluorescence | Control |
| Control12 | 53 | Woman | Immunofluorescence | Control |
| DC1 | 47 | Man | Proteomics | DC |
| DC2 | 52 | Man | Proteomics | DC |
| DC3 | 68 | Woman | Proteomics | DC |
| DC4 | 70 | Man | Proteomics | DC |
| DC5 | 75 | Man | Proteomics | DC |
| DC6 | 46 | Man | Proteomics | DC |
| DC7 | 34 | Man | Proteomics | DC |
| DC8 | 42 | Man | Proteomics | DC |
| DC9 | 73 | Woman | Proteomics | DC |

## Slide 3
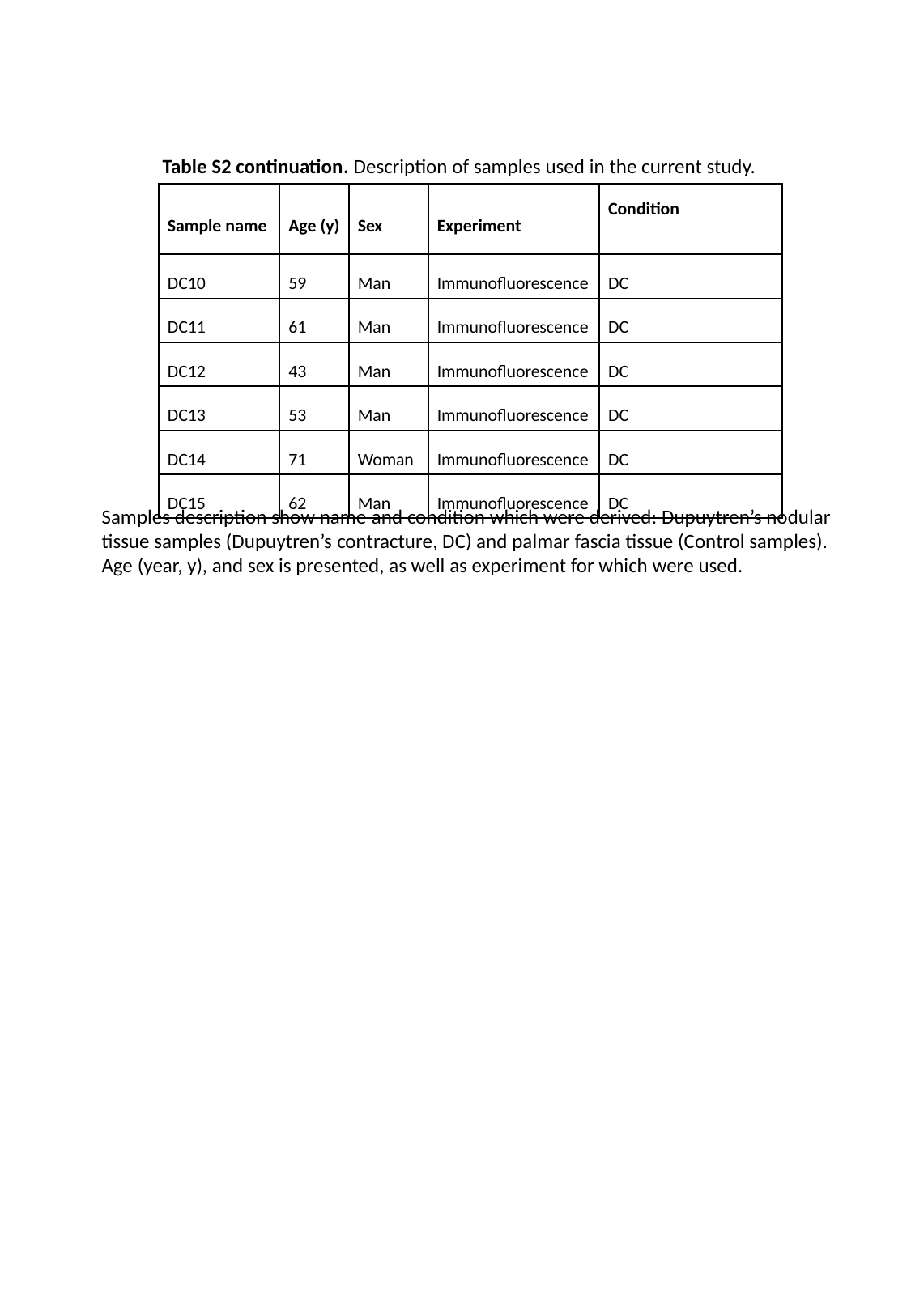

Table S2 continuation. Description of samples used in the current study.
| Sample name | Age (y) | Sex | Experiment | Condition |
| --- | --- | --- | --- | --- |
| DC10 | 59 | Man | Immunofluorescence | DC |
| DC11 | 61 | Man | Immunofluorescence | DC |
| DC12 | 43 | Man | Immunofluorescence | DC |
| DC13 | 53 | Man | Immunofluorescence | DC |
| DC14 | 71 | Woman | Immunofluorescence | DC |
| DC15 | 62 | Man | Immunofluorescence | DC |
Samples description show name and condition which were derived: Dupuytren’s nodular tissue samples (Dupuytren’s contracture, DC) and palmar fascia tissue (Control samples). Age (year, y), and sex is presented, as well as experiment for which were used.

## Slide 4
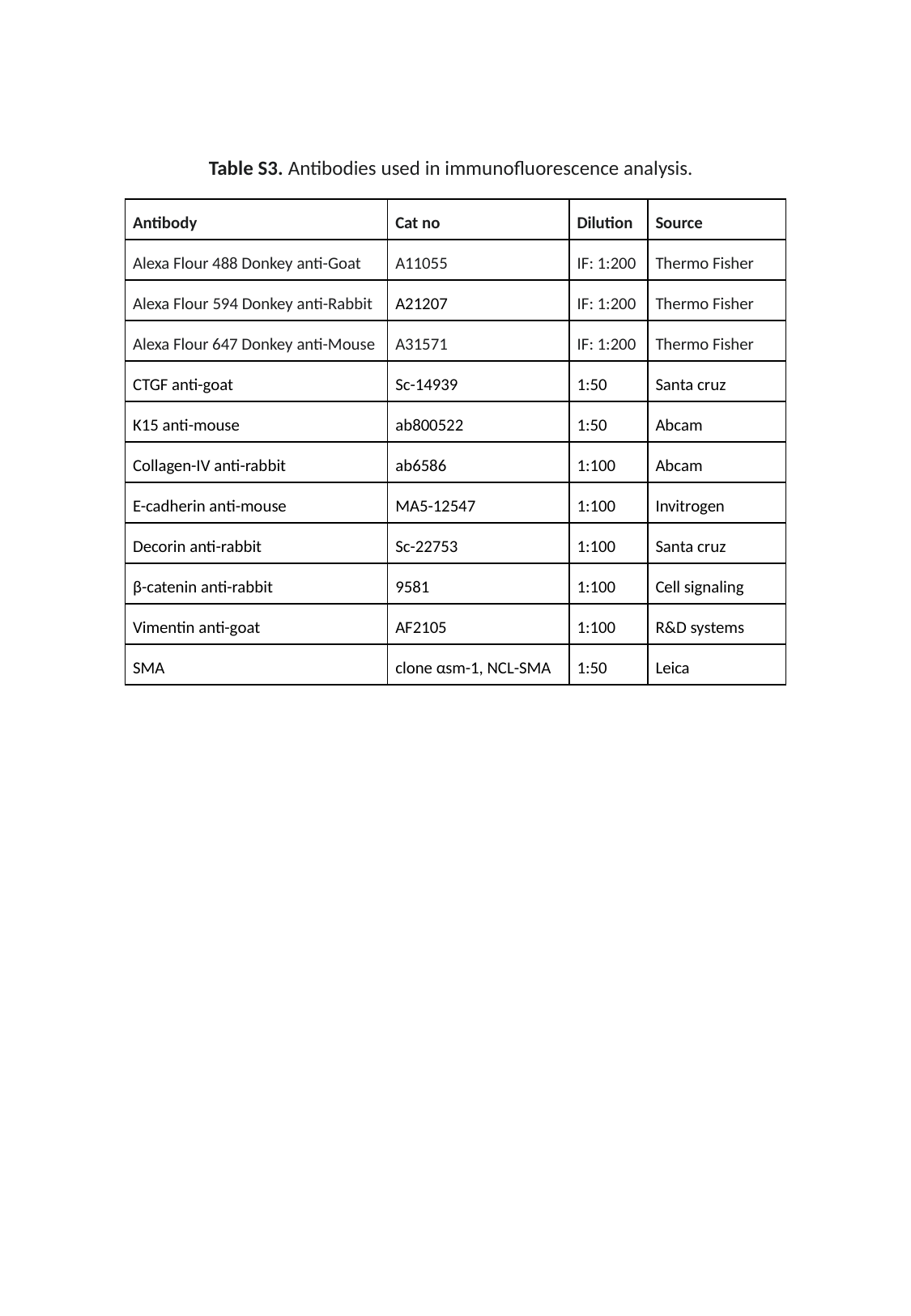

Table S3. Antibodies used in immunofluorescence analysis.
| Antibody | Cat no | Dilution | Source |
| --- | --- | --- | --- |
| Alexa Flour 488 Donkey anti-Goat | A11055 | IF: 1:200 | Thermo Fisher |
| Alexa Flour 594 Donkey anti-Rabbit | A21207 | IF: 1:200 | Thermo Fisher |
| Alexa Flour 647 Donkey anti-Mouse | A31571 | IF: 1:200 | Thermo Fisher |
| CTGF anti-goat | Sc-14939 | 1:50 | Santa cruz |
| K15 anti-mouse | ab800522 | 1:50 | Abcam |
| Collagen-IV anti-rabbit | ab6586 | 1:100 | Abcam |
| E-cadherin anti-mouse | MA5-12547 | 1:100 | Invitrogen |
| Decorin anti-rabbit | Sc-22753 | 1:100 | Santa cruz |
| β-catenin anti-rabbit | 9581 | 1:100 | Cell signaling |
| Vimentin anti-goat | AF2105 | 1:100 | R&D systems |
| SMA | clone αsm-1, NCL-SMA | 1:50 | Leica |

## Slide 5
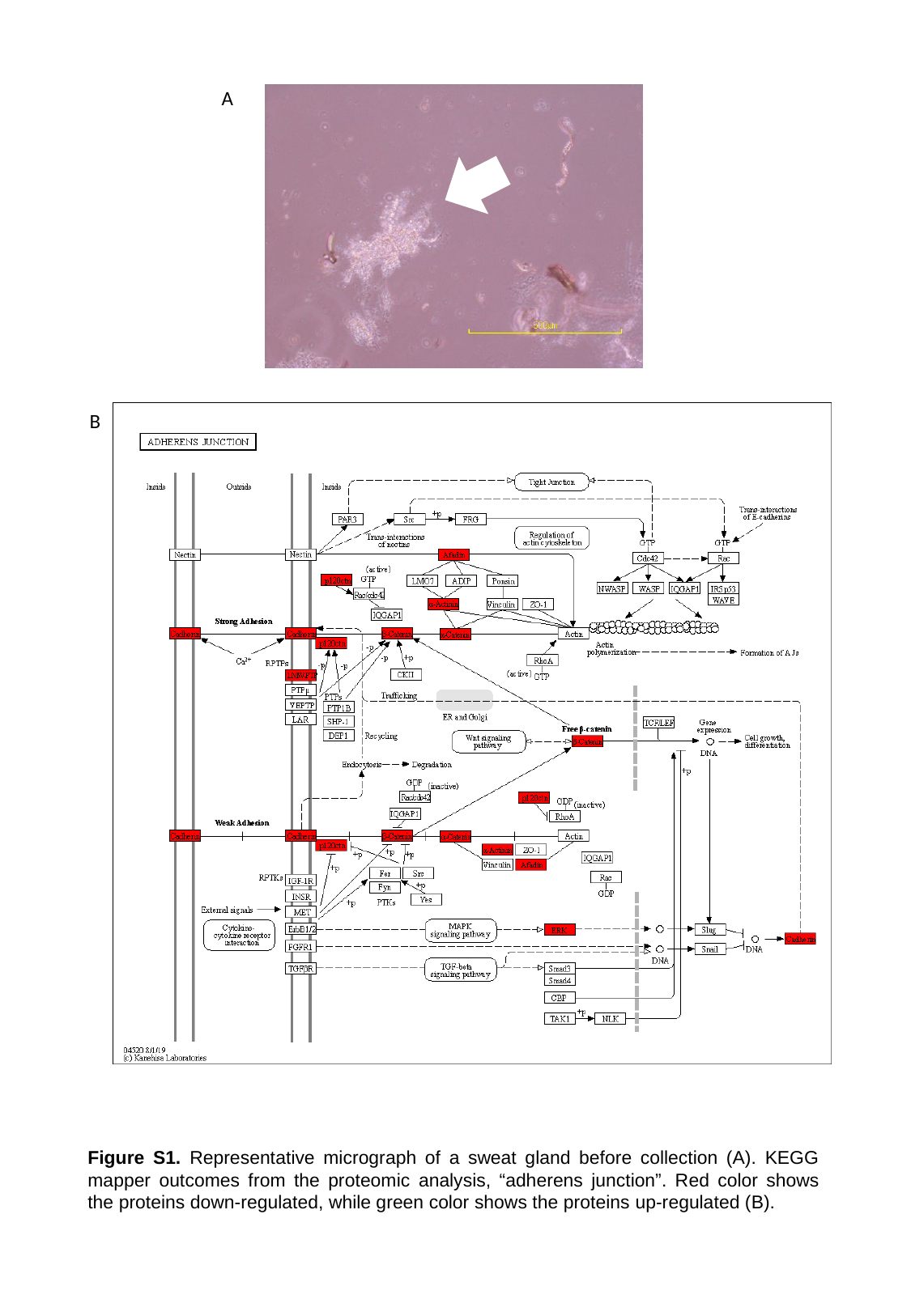

A
B
Figure S1. Representative micrograph of a sweat gland before collection (A). KEGG mapper outcomes from the proteomic analysis, “adherens junction”. Red color shows the proteins down-regulated, while green color shows the proteins up-regulated (B).

## Slide 6
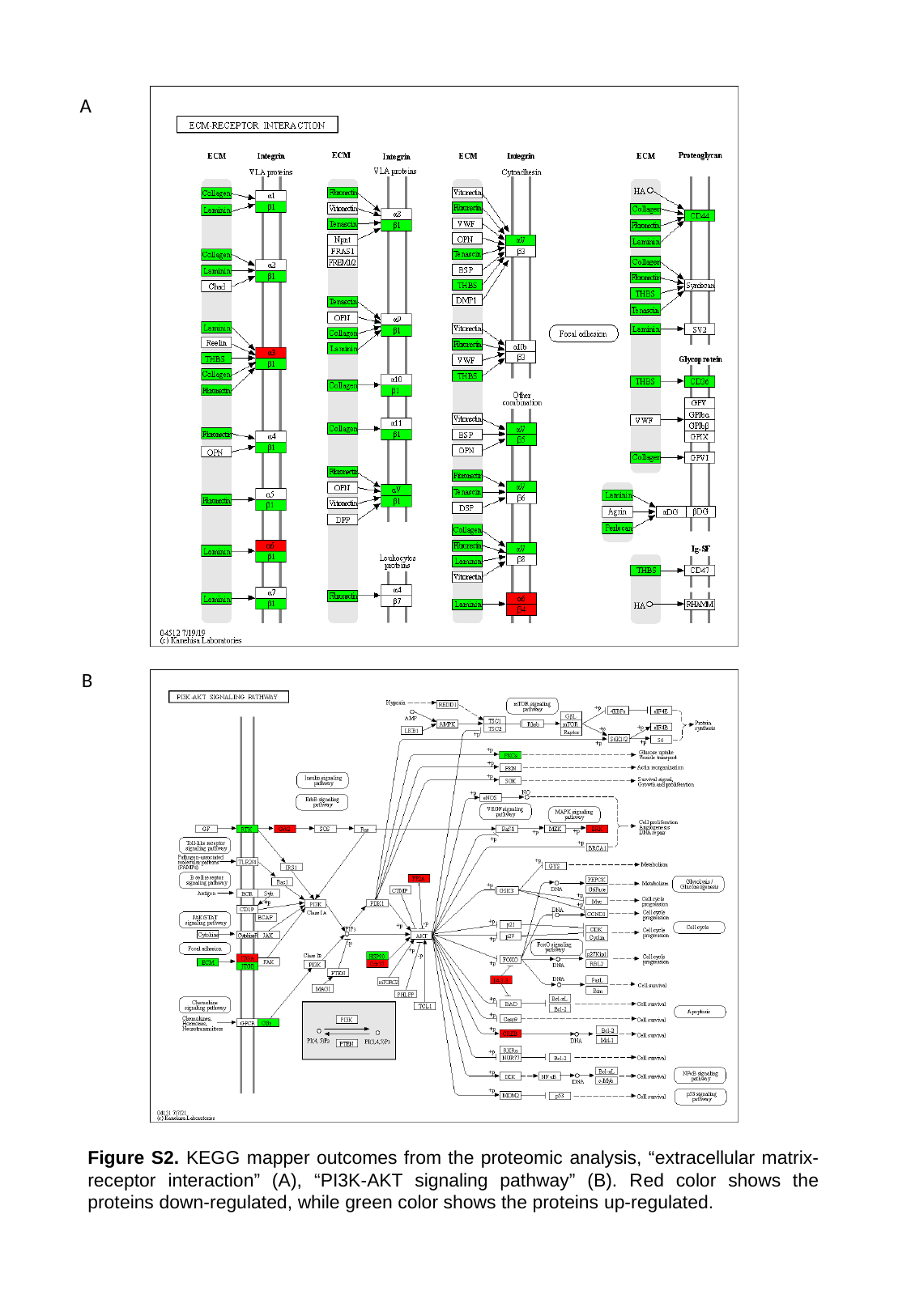

A
B
Figure S2. KEGG mapper outcomes from the proteomic analysis, “extracellular matrix-receptor interaction” (A), “PI3K-AKT signaling pathway” (B). Red color shows the proteins down-regulated, while green color shows the proteins up-regulated.

## Slide 7
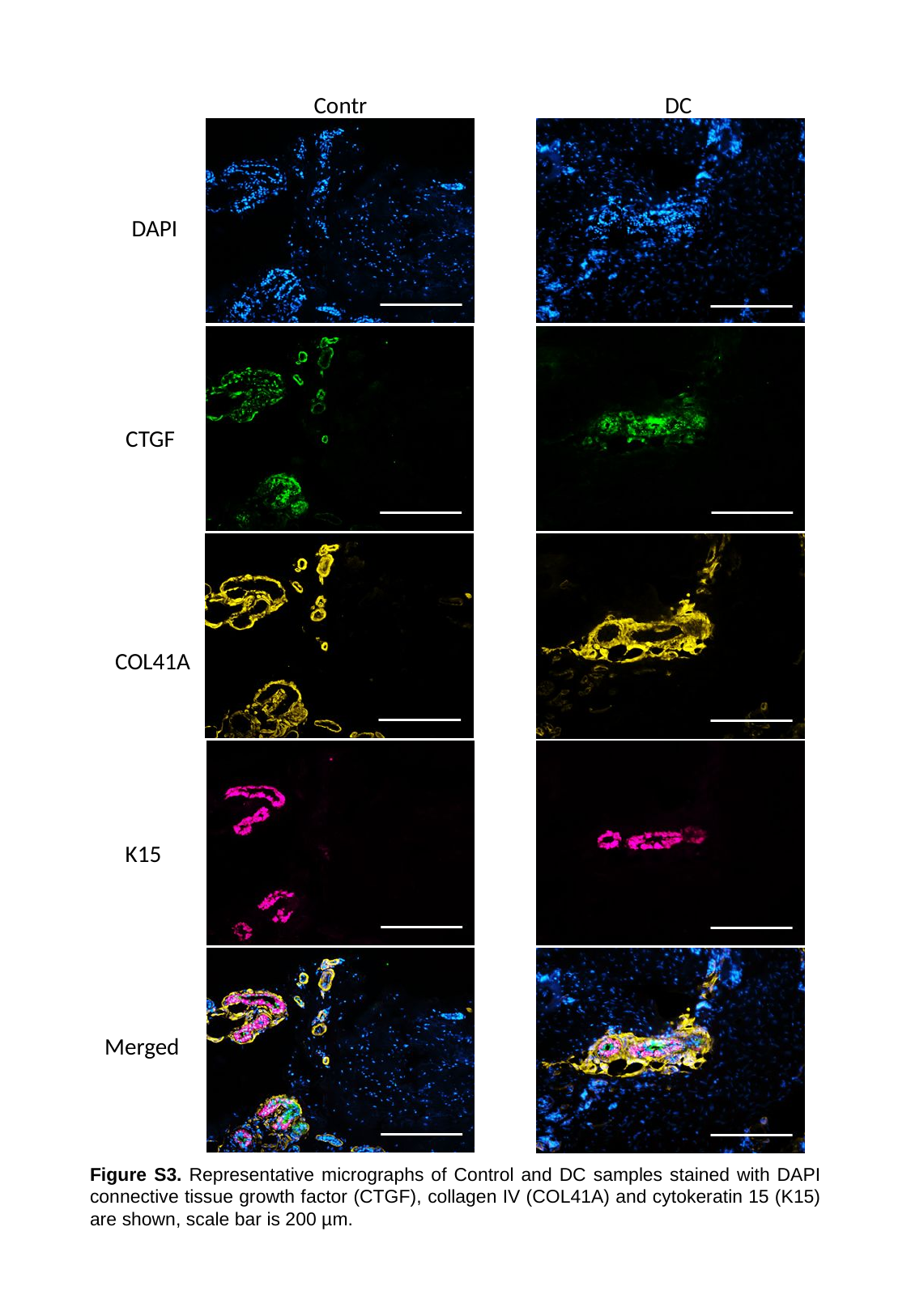

DC
Control
DAPI
CTGF
COL41A
K15
Merged
Figure S3. Representative micrographs of Control and DC samples stained with DAPI connective tissue growth factor (CTGF), collagen IV (COL41A) and cytokeratin 15 (K15) are shown, scale bar is 200 µm.

## Slide 8
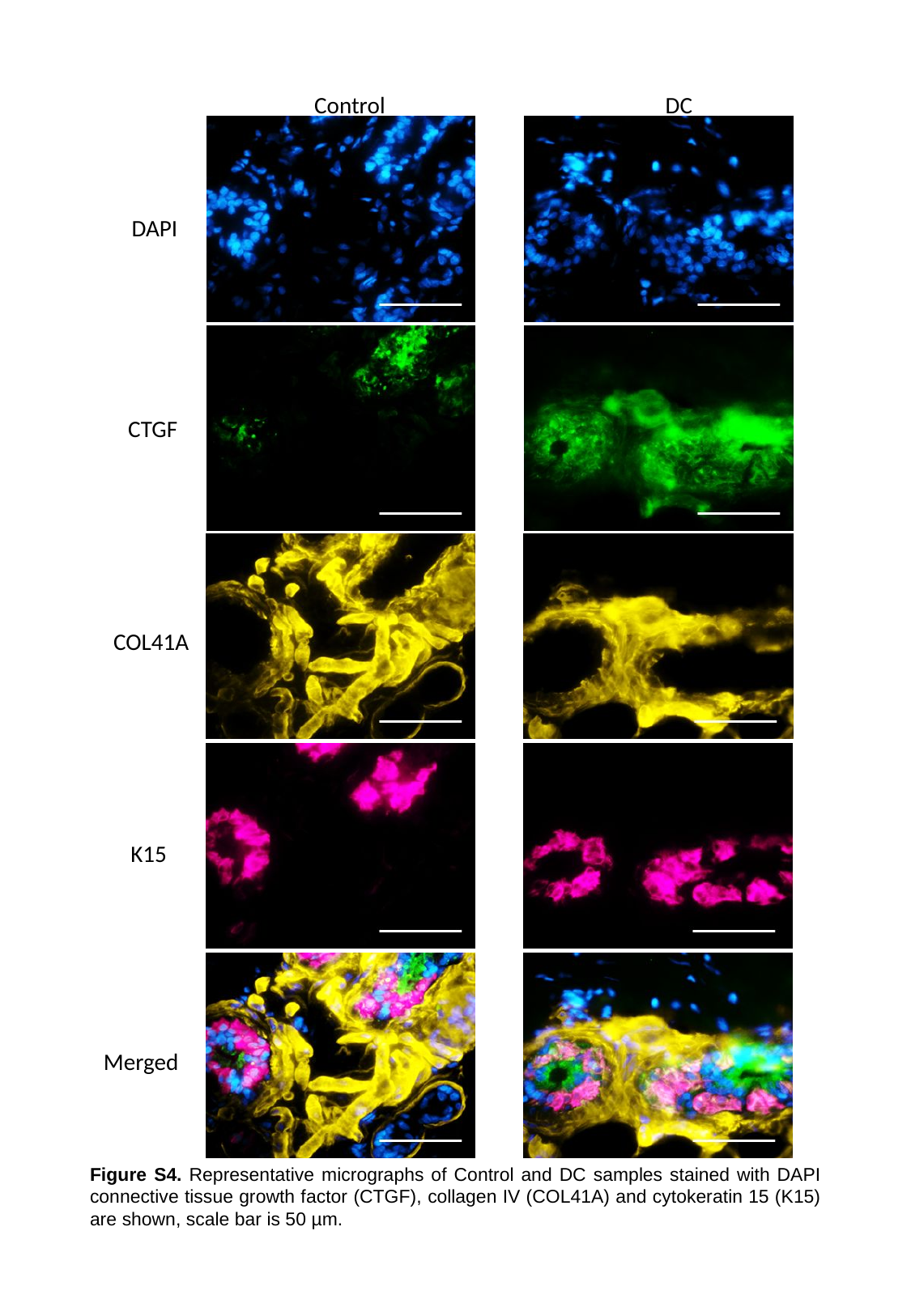

DC
Control
DAPI
CTGF
COL41A
K15
Merged
Figure S4. Representative micrographs of Control and DC samples stained with DAPI connective tissue growth factor (CTGF), collagen IV (COL41A) and cytokeratin 15 (K15) are shown, scale bar is 50 µm.

## Slide 9
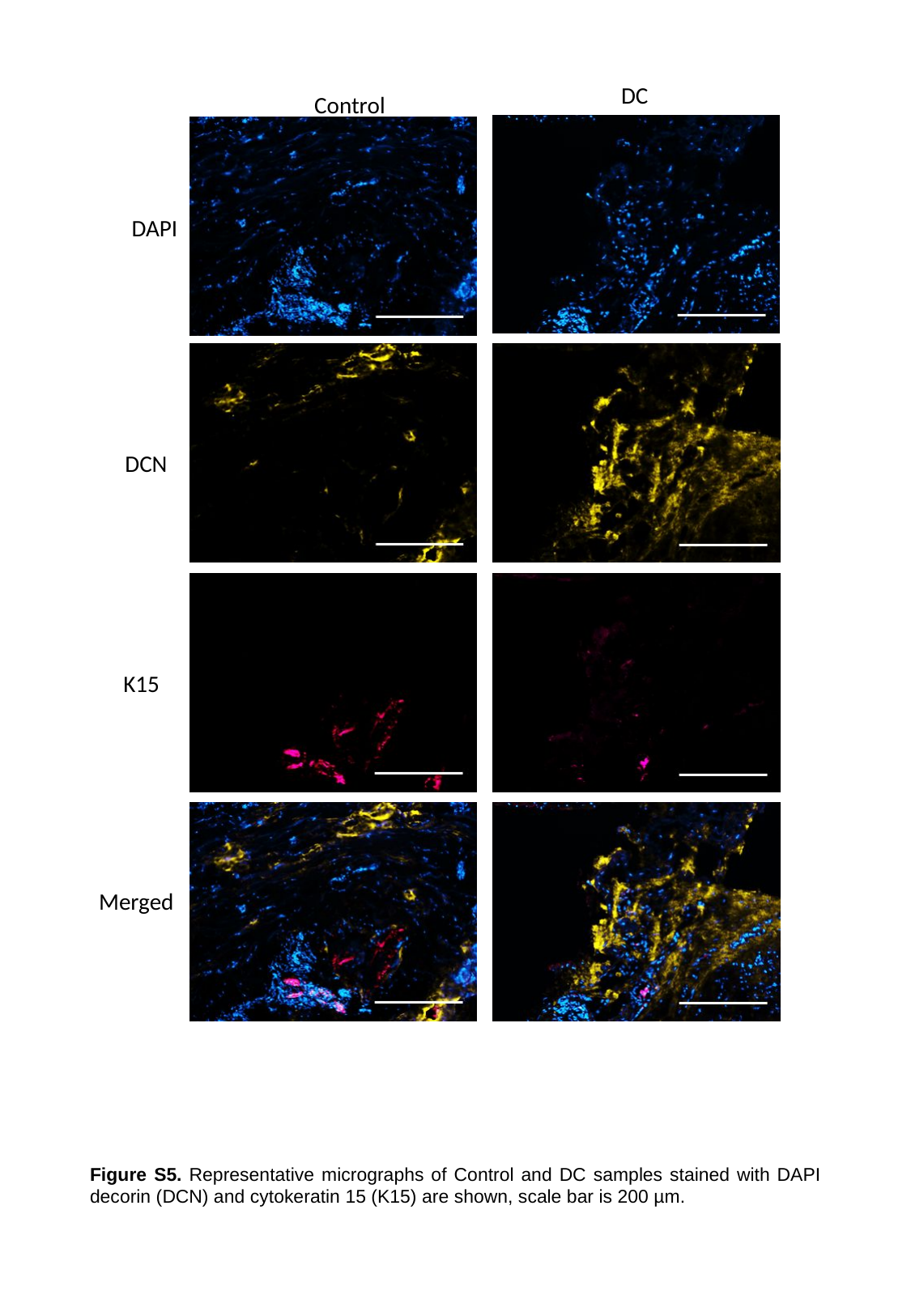

DC
Control
DAPI
DCN
K15
Merged
Figure S5. Representative micrographs of Control and DC samples stained with DAPI decorin (DCN) and cytokeratin 15 (K15) are shown, scale bar is 200 µm.

## Slide 10
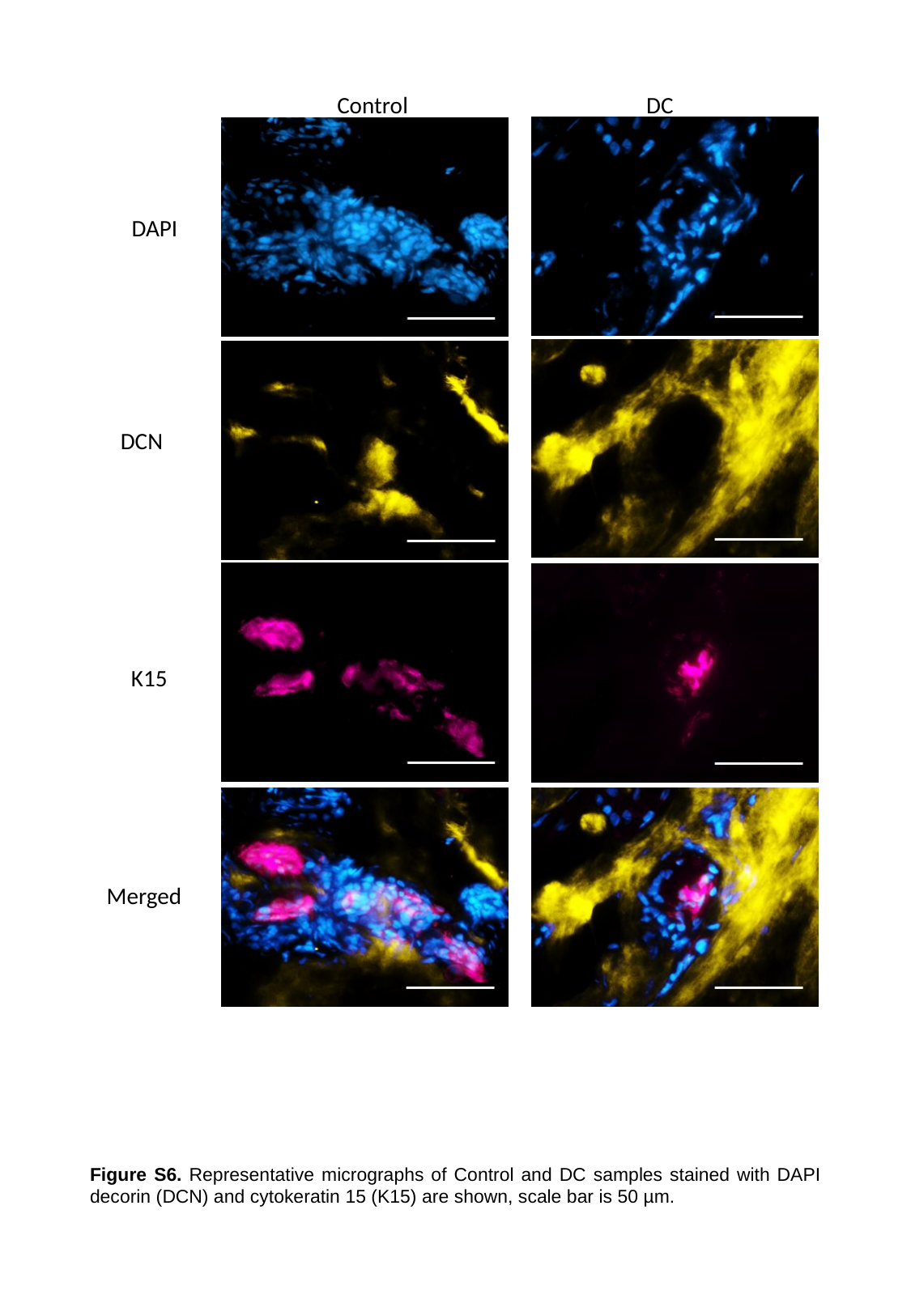

DC
Control
DAPI
DCN
K15
Merged
Figure S6. Representative micrographs of Control and DC samples stained with DAPI decorin (DCN) and cytokeratin 15 (K15) are shown, scale bar is 50 µm.

## Slide 11
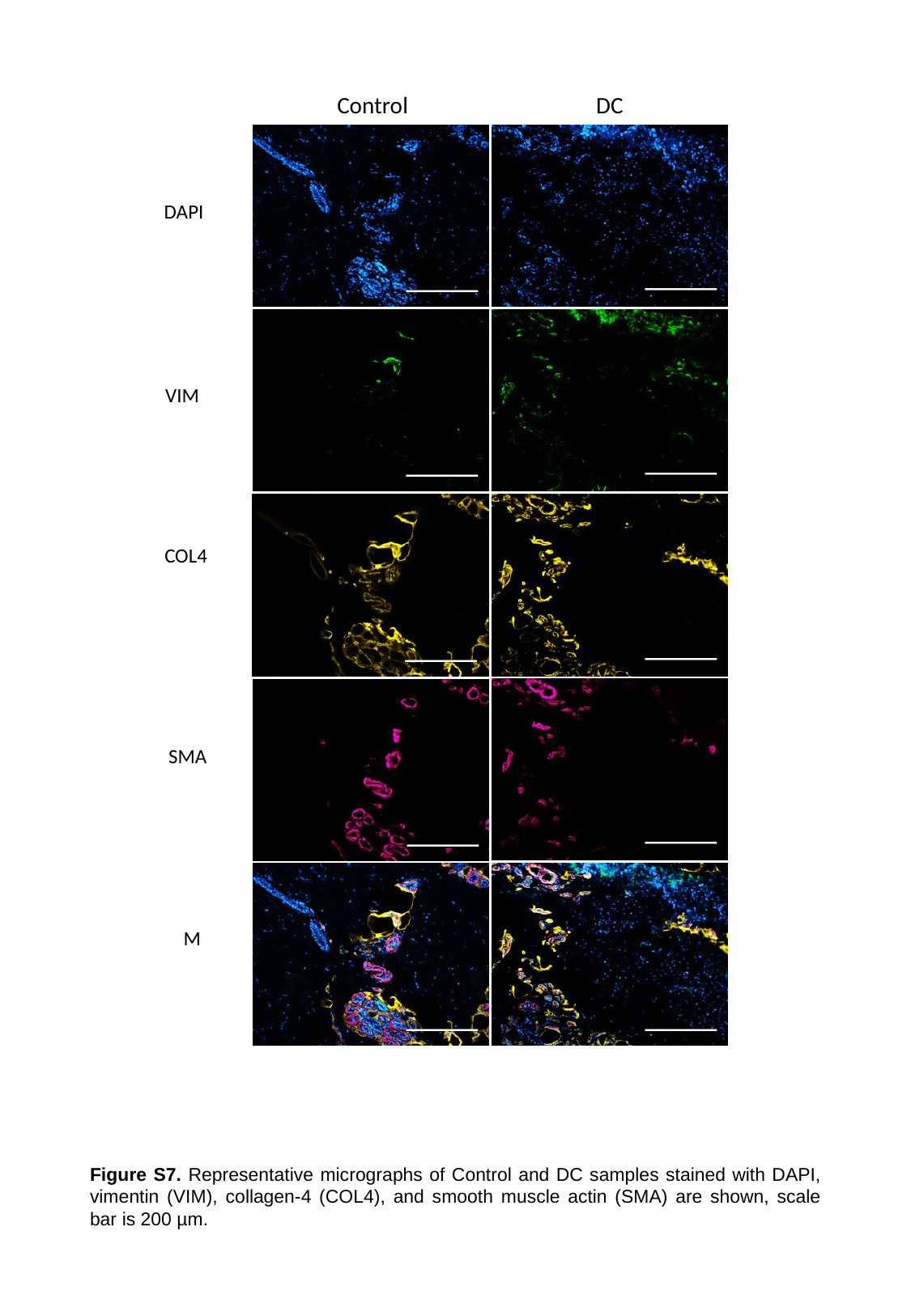

DC
Control
DAPI
VIM
COL4
SMA
M
Figure S7. Representative micrographs of Control and DC samples stained with DAPI, vimentin (VIM), collagen-4 (COL4), and smooth muscle actin (SMA) are shown, scale bar is 200 µm.

## Slide 12
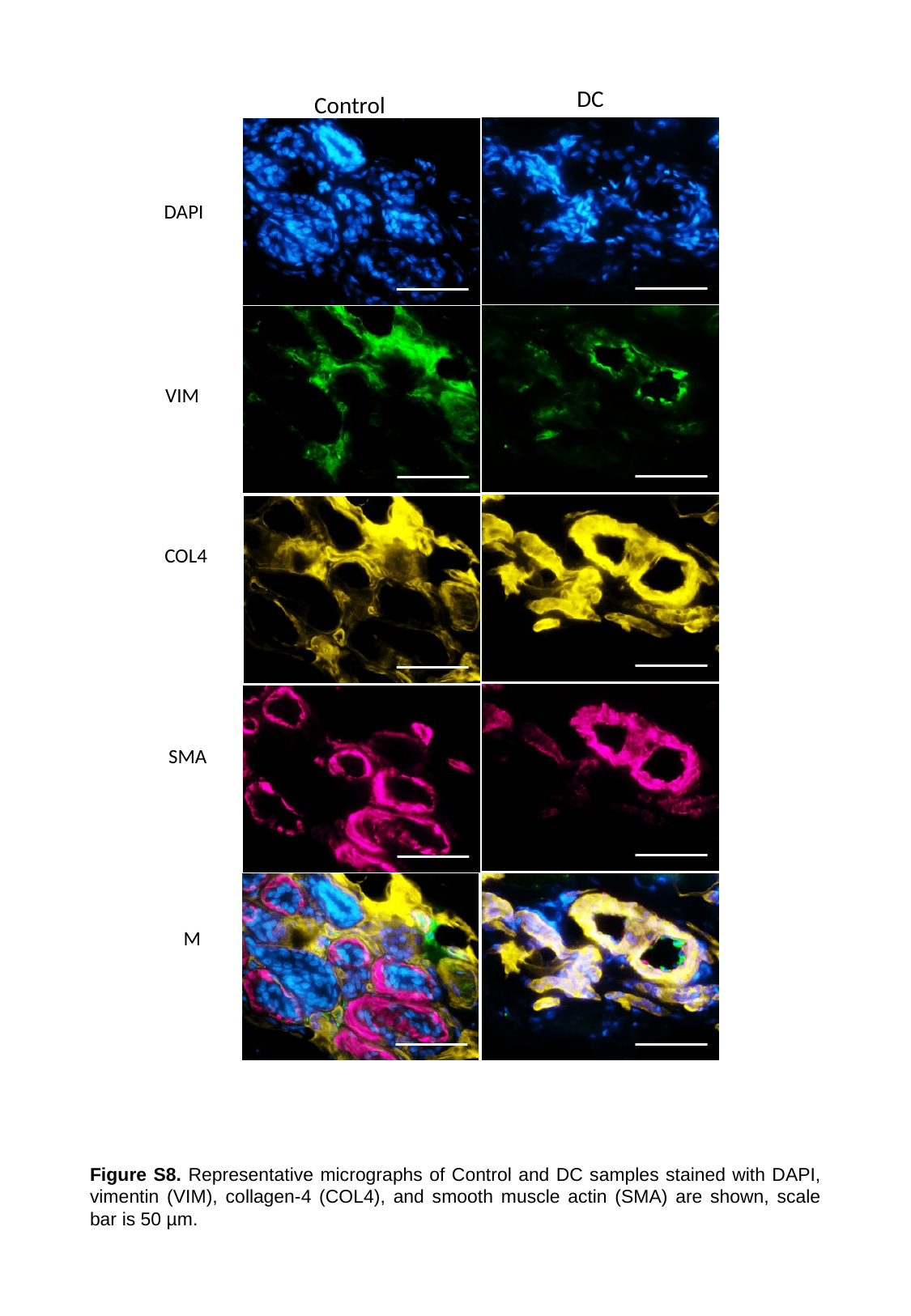

DC
Control
DAPI
VIM
COL4
SMA
M
Figure S8. Representative micrographs of Control and DC samples stained with DAPI, vimentin (VIM), collagen-4 (COL4), and smooth muscle actin (SMA) are shown, scale bar is 50 µm.

## Slide 13
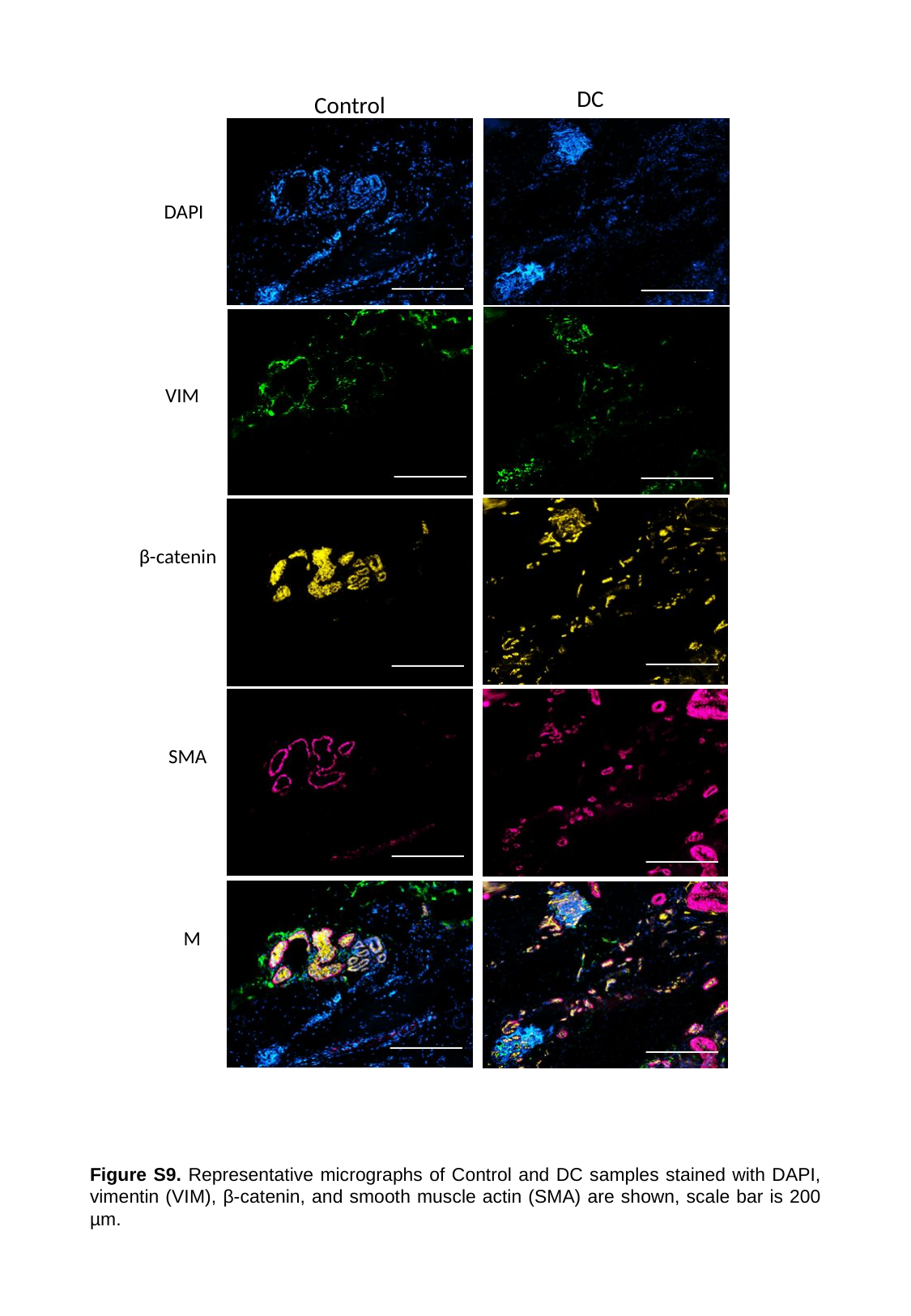

DC
Control
DAPI
VIM
β-catenin
SMA
M
Figure S9. Representative micrographs of Control and DC samples stained with DAPI, vimentin (VIM), β-catenin, and smooth muscle actin (SMA) are shown, scale bar is 200 µm.

## Slide 14
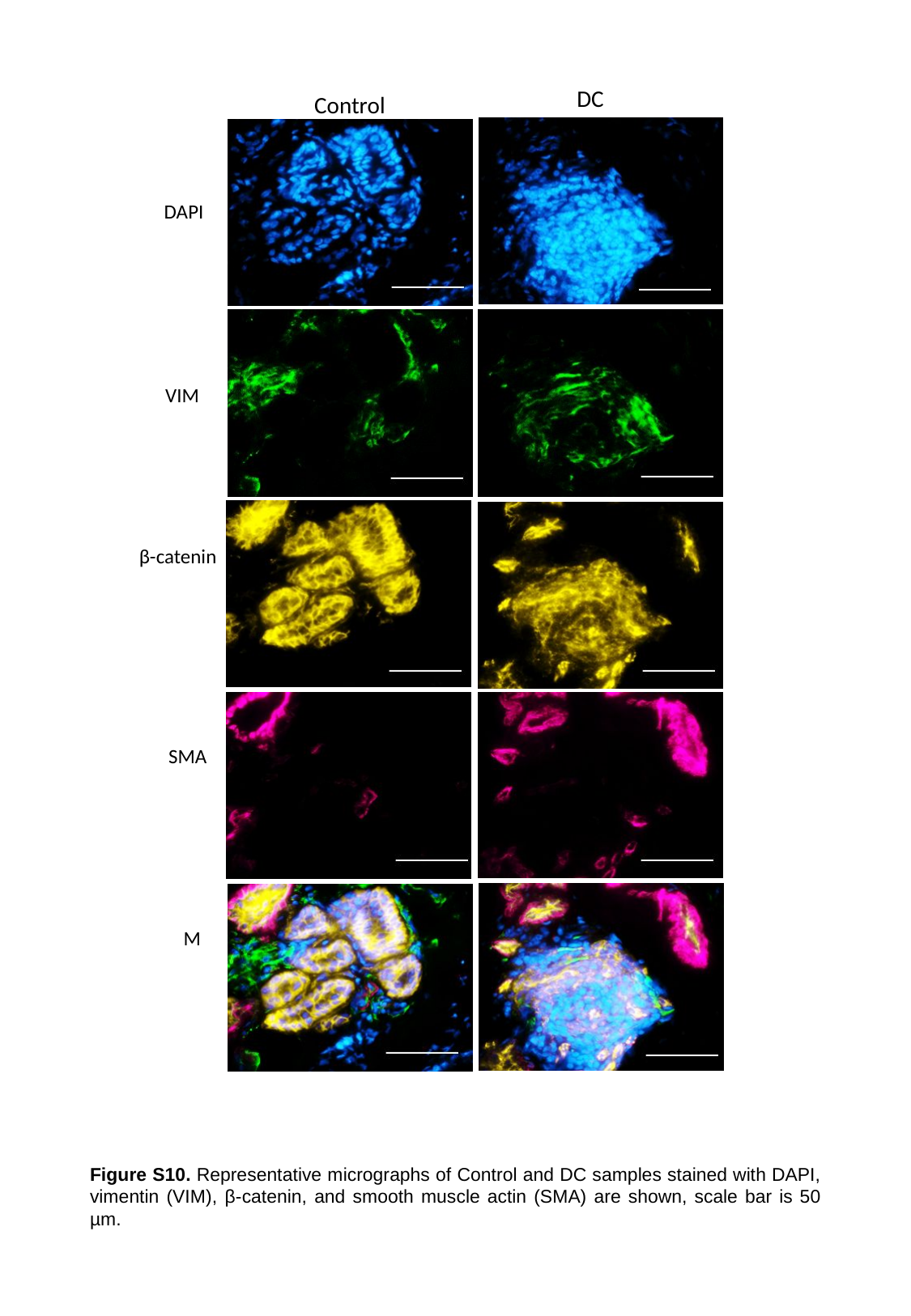

DC
Control
DAPI
VIM
β-catenin
SMA
M
Figure S10. Representative micrographs of Control and DC samples stained with DAPI, vimentin (VIM), β-catenin, and smooth muscle actin (SMA) are shown, scale bar is 50 µm.
